# Detection of EGFR Mutations in Plasma cfDNA and Paired CTCs of NSCLC Patients before and after Osimertinib Therapy Using Crystal Digital PCR

**DOI:** 10.3390/cancers13112736

**Published:** 2021-05-31

**Authors:** Aliki Ntzifa, Athanasios Kotsakis, Vassilis Georgoulias, Evi Lianidou

**Affiliations:** 1 Analysis of Circulating Tumor Cells Lab, Lab of Analytical Chemistry, Department of Chemistry, National and Kapodistrian University of Athens, 15771 Athens, Greece; alntzi@chem.uoa.gr; 2 Department of Medical Oncology, General University Hospital of Larissa, 41110 Larissa, Greece; thankotsakis@uth.gr; 3 Hellenic Oncology Research Group (HORG), 11471 Athens, Greece; georgulv@otenet.gr

**Keywords:** liquid biopsy, circulating tumor DNA (ctDNA), circulating tumor cells (CTC), crystal digital PCR, EGFR mutations, osimertinib

## Abstract

**Simple Summary:**

Liquid biopsy is a useful tool during longitudinal monitoring of NSCLC patients and requires highly sensitive and reliable technologies for accurate detection of genomic alterations. We evaluated and used crystal digital PCR to detect and quantify EGFR mutations in plasma cfDNA and paired CTCs of NSCLC patients before treatment with osimertinib and at progression of disease.

**Abstract:**

Circulating tumor DNA (ctDNA) analysis has clinical utility in EGFR mutant NSCLC. Circulating tumor cells (CTCs) consist a unique source of information at the cellular level. Digital PCR (dPCR) is a valuable tool for accurate and valid analysis of gene mutations in liquid biopsy analysis. In the present study we detected EGFR mutations in ctDNA and paired CTCs under osimertinib therapy at two time points using crystal dPCR and the naica^®^ system (Stilla Technologies). We quantified mutation allele frequencies (MAF) of EGFR mutations in 91 plasma cfDNA samples of 48 EGFR mutant NSCLC patients and in 64 matched CTC-derived genomic DNA samples, and the FDA-cleared cobas^®^ EGFR mutation test in 80 identical plasma samples. Direct comparison between crystal dPCR and the cobas EGFR assay revealed a high concordance for all EGFR mutations. Our comparison of crystal dPCR results in ctDNA with the corresponding primary tissue has shown a strong correlation. EGFR mutations analysis in paired CTC-derived gDNA revealed a high heterogeneity. Crystal dPCR offers the unique advantages of high analytical sensitivity, precision, and accuracy for detecting and quantifying multiple EGFR mutations in plasma cfDNA and CTCs of NSCLC patients.

## 1. Introduction

The non-small cell lung cancer (NSCLC) treatment landscape has changed through the last two decades [1] and several clinical trials have revealed significantly improved progression-free survival (PFS) and overall survival (OS) [2,3,4,5]. Thus, molecularly targeted therapies have gradually replaced chemotherapy. However, the progression of disease due to the emergence of different resistance mechanisms was always a challenge to overcome. The most common resistance mechanism to 1st or 2nd generation inhibitors of the tyrosine kinase domain of epidermal growth factor receptor (EGFR TKIs) is the presence of exon 20 T790M EGFR mutation [6] and was treated with the administration of osimertinib [7], a 3rd generation EGFR TKI which was proved to be effective against both T790M and sensitizing mutations [8]. Despite the rationale of EGFR TKI treatment sequence, in 2018, the US Food and Drug Administration (FDA) approved osimertinib as a 1st line treatment for patients with metastatic EGFR mutant NSCLC previously untreated based on the promising results of the FLAURA study that demonstrated superior efficacy of osimertinib over 1st and 2nd generation EGFR TKIs [9,10].

Longitudinal monitoring of patients during disease and their treatment management requires serial tumor genotyping to follow genomic alterations that are responsible for disease progression and therapy resistance [11]. Liquid biopsy as a minimally invasive method and complementary to traditional tissue biopsy consists a more feasible approach to track tumor evolution [12]. In case of NSCLC management, circulating tumor DNA (ctDNA) genotyping has been successfully integrated in the clinical setting [13,14]. Since FDA’s first approval of the cobas^®^ EGFR CE-IVD Mutation Test (Roche Molecular Systems, Inc., Pleasanton, CA, USA) in 2016, as a companion diagnostic for osimertinib [15], another liquid biopsy test, the Guardant360 CDx (Guardant Health, Inc., Redwood City, CA, USA), has been recently verified by FDA for EGFR mutant NSCLC patients who may benefit from treatment with osimertinib [16,17]. On the other hand, circulating tumor cell (CTC) analysis entails various technical challenges that hamper its extensive use in clinical practice, up to date [18,19]. Nevertheless, limited but remarkable studies have demonstrated that the detection of EGFR mutations in CTCs of NSCLC patients could mirror tumor heterogeneity [20] and the emergence of clonal evolution under treatment selective pressure [21,22,23,24].

Hence, there are many crucial aspects to consider for the integration of liquid biopsy into clinical routine. Validation of the preanalytical procedures through well-established protocols is of utmost importance for ensuring the highest quality and quantity of circulating biomarkers. Several notable studies on the preanalytical factors, from blood collection and transportation to cell-free DNA (cfDNA) extraction and quantification, that affect liquid biopsy analysis have already been made [25,26,27] and recommendations for the quality control of the whole procedure have been proposed [28].

Once the primary step of the preanalytical phase is successfully implemented, another key factor is the selection of the appropriate methodology for downstream molecular analysis. Moving from relative to absolute quantification gives us the opportunity to detect molecular alterations at extremely low limits of detection, an essential for reliable liquid biopsy analyses [29]. Thus far, molecular analysis based on PCR methods has improved through recent years with the emergence of cutting-edge technologies such as digital PCR (dPCR) [29] and BEAMing [30]. dPCR consists a breeding ground for molecular analysis in the field of oncology by providing improved precision, increased dynamic range and analytical sensitivity while detecting rare events [31]. For that purpose, several digital PCR platforms are designed to combine the unique features of dPCR with the capacity of using multiple detection channels [32,33,34,35].

The aim of the current study was to detect EGFR mutations in plasma cfDNA of NSCLC patients that are longitudinally monitored during osimertinib therapy before the initiation of therapy and at progression of disease using the crystal dPCR technology and the naica^®^ system. In parallel, the presence of EGFR mutations was assessed in identical plasma samples analyzed with the FDA approved cobas^®^ technology and in paired CTC enriched samples isolated during the same blood draw with crystal dPCR. We further compared our findings with those derived from the analysis of the corresponding primary tissues. Finally, we quantified EGFR mutation allele frequencies (MAFs) in cfDNA and paired CTC fractions before therapy initiation and at PD. All procedures from preanalytical to analytical phase were implemented within strict quality control requirements. 

## 2. Materials and Methods

### 2.1. Patients

Forty-eight patients with histologically or cytologically documented EGFR mutated lung adenocarcinomas previously treated with 1st and/or 2nd generation EGFR TKIs were included in a multicenter Phase II clinical study [ClinicalTrials.gov, accessed date 5 April 2021, number: NCT02771314, registration date: 13 May 2016 and EudraCT number: 2016-001335-12, registration date: 13 April 2016] conducted by the Hellenic Oncology Research Group (HORG). Osimertinib (AZD9291; Astra Zeneca, Cambridge, UK) was administered as a 2nd line treatment in 15/48 (52.1%) and as 3rd line in 23/48 (47.9%) upon their progression of disease with EGFR TKIs. Their median age was 66.5 (range: 43–87 years) and 35/48 (72.9%) were female. In addition, a group of 10 healthy donors (HD) was used as a control group. The study has been approved by the National Drug Administration of Greece (EOF), the National Ethics Committee (35/00-03/16, 35/03-11/16) and the Institutional Ethical Committees of the HORG’s participating centers. The study was conducted in accordance with the Declaration of Helsinki. All patients and HD gave their written informed consent to participate. 

### 2.2. Peripheral Blood Sampling and Processing

Peripheral blood (PΒ) was obtained at two time points: (a) before the treatment initiation with osimertinib (baseline; *n* = 48 samples) and (b) at the time of disease progression (PD: *n* = 43 samples). At the time of analysis, four patients were still under osimertinib therapy. In total, 91 patient samples and 10 HD samples were further processed and analyzed following exactly the same steps. PB (15 mL) was collected in tubes containing ethylenediaminetetraacetic acid (EDTA) as anticoagulant, after discarding the first 5 mL of blood draw to avoid contamination of skin epithelial cells. Blood samples were centrifuged at 530× *g* for 10 min at room temperature (RT) and plasma was separated from buffy coat and erythrocytes. Plasma samples were then subjected to a second centrifugation at 16,000× *g* for 10 min at RT and transferred to a new tube. Aliquots of identical plasma samples from every single blood sampling were kept at −80 °C until cfDNA extraction.

### 2.3. cfDNA Extraction

2 mL of plasma was used for the cfDNA extraction using the IDXtract kit (ID-Solutions, Grabels, France), a silica membrane nucleic acid extraction system, according to the manufacturer’s instructions. The efficiency of extraction process was assessed by adding an exogenous internal extraction control (ICE) in every plasma sample prior to extraction and by using a negative and a target positive control extracted in the same way as plasma samples. The final elution volume of extracted cfDNA was 65 μL. In parallel, another identical aliquot of 2 mL plasma for each sample was extracted using the cobas^®^ cfDNA Sample Preparation Kit (Roche Molecular Systems, Inc., Pleasanton, CA, USA), a system that uses columns with a glass fiber filter insert, according to manufacturer’s instructions, to a final volume of 100 μL of elution buffer.

### 2.4. CTC Enrichment and Genomic DNA Extraction

After separating plasma from blood cells, an equal volume of removed plasma was replaced by adding phosphate buffered saline (PBS, pH 7.3) into the cell pellet and then samples were proceeded for CTC enrichment in the size-based microfluidic device, Parsortix™ (ANGLE plc, Guildford, UK), using a 6.5 μm separation cassette, as previously described [36]. Captured cells were harvested in 200 μL of PBS, genomic DNA (gDNA) was extracted from the CTC fraction using the TRIZOL-LS reagent (Thermo Fisher Scientific, Inc., Waltham, MA, USA) as previously described [37] and isolated gDNA was dissolved in a final volume of 20 μL of 8 mmol/L NaOH. DNA concentration was measured in a NanoDrop-1000 spectrophotometer (Thermo Fisher Scientific, Inc., Waltham, MA, USA) and calibrated with the recommended CF-1 standard solution.

### 2.5. Whole Genome Amplification of Genomic DNA

3–5 μL of gDNA, extracted from the enriched CTC fractions, was amplified using the *Ampli*1™ Whole Genome Amplification (WGA) kit (Menarini Silicon Biosystems, Castel Maggiore, BO, Italy). The WGA procedure was optimized for using gDNA derived from CTC fraction following the protocol steps according to manufacturer’s instructions except for the lysis step that had been already implemented during gDNA extraction with TRIZOL-LS. All samples were checked for their DNA integrity after WGA and prior to mutational analysis, by amplifying a region of beta-actin (*β*-*actin*) gene using RT-qPCR.

### 2.6. cfDNA Analysis

#### 2.6.1. Crystal Digital PCR

All cfDNA samples (*n* = 91) extracted with IDXtract kit were processed for the quantification of cfDNA using the IDQUANTd kit (ID-Solutions, Grabels, France) by Crystal digital PCR™, with the naica^®^ system (Stilla Technologies, Villejuif, France). The kit offers the simultaneous quantification of total cfDNA and the ICE to assess the extraction process and to standardize the concentration of each sample. The internal control is also used to standardize inter-assay fluctuations for the same patient during longitudinal monitoring by using the following Equation (1):

[cfDNA (copies/μL)/ICE (copies/μL)] × 1/extracted volume of DNA(μL)(1)

In this step, crystal dPCR also gives us the opportunity to assess the quality of samples by two critical factors: (a) the number of generated droplets and (b) the number of saturated objects that denote the possible presence of PCR inhibitors. After quantification, the cfDNA samples were analyzed by Crystal digital PCR™ and the naica^®^ system for the presence of mutations in exons 18, 19, 20 and 21 of the EGFR gene (Supplementary Table S1) using three different multiplex digital PCR kits (ID-Solutions): IDEGFR SENSI-50, IDEGFR RESIST-50 and IDEGFR RARE-50 according to manufacturer’s instructions. Moreover, using crystal dPCR we quantified EGFR %MAF in all samples. 

#### 2.6.2. Real-Time PCR

All cfDNA samples (*n* = 80) extracted with cobas^®^ cfDNA Sample Preparation Kit were further analyzed by the cobas^®^ EGFR Mutation Test v2, a real-time PCR test that identifies 42 mutations in exons 18, 19, 20 and 21 of the EGFR gene (Supplementary Table S1) in the cobas^®^ z 480 analyzer (Roche Molecular Systems, Inc.). Our lab is accredited according to ISO-15189 standard for EGFR testing in plasma samples with cobas^®^ technology [38].

### 2.7. CTC Analysis with Crystal Digital PCR

We applied for the first time crystal dPCR to detect EGFR mutations in gDNA isolated from paired CTC-derived gDNA (*n* = 64). Since the ID-Solutions kits are used for EGFR mutation detection in plasma cfDNA samples, we used the human cell line NCI-H1975 to optimize detection assays in gDNA derived from matched CTC fractions (*n* = 64). After WGA, 15 μL of amplified DNA in a dilution 1:100 was used for the detection of EGFR mutations by crystal digital PCR™ in the naica^®^ system.

### 2.8. Statistical Analysis

Statistical analysis was performed using SPSS Statistics 26.0 (IBM Corp., Armonk, NY, USA). The chi-square test of independence and the Mann Whitney test were used to compare the different groups. The Cohen’s kappa index was used to compare the two methods. Progression free survival (PFS) was defined as the time from treatment with osimertinib to the first documented progression, death from any cause or last contact, whichever occurred first. Survival distributions were estimated using the Kaplan-Meier method and compared across groups with the log-rank test. All statistical tests were two sided, and *p*-values less than 0.05 were considered statistically significant. Levey-Jennings graphs, created by using MS Office Excel (Microsoft Corporation, Redmond, WA, USA), were used to monitor and evaluate the extraction process. 

## 3. Results

**Figure 1:**
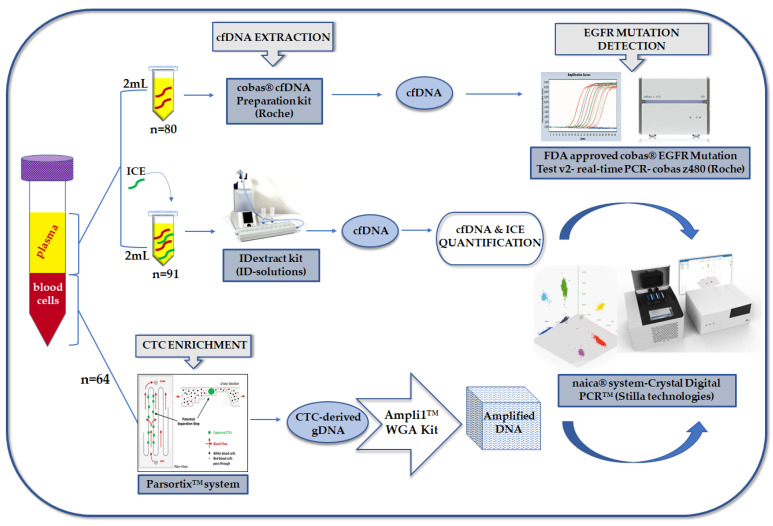
The schematic flowchart of the study.

The outline of the study is shown in Figure 1.

### 3.1. Crystal dPCR for the Evaluation of Preanalytical Procedure

**Figure 2:**
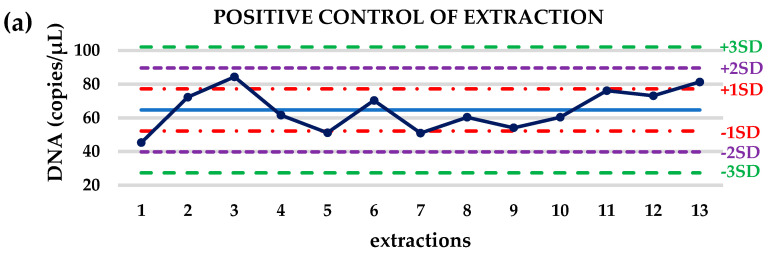

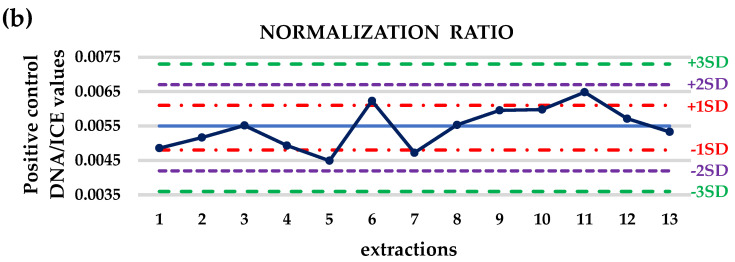
Levey-Jennings graphs for the evaluation of extraction process based on (**a**) DNA concentration (copies/μL) of the target positive control of extraction and on (**b**) normalized positive control DNA/ICE values.

During the extraction process of plasma samples, internal extraction control (ICE) was added in every sample to assess the efficiency of the extraction. Furthermore, during every single extraction experiment a negative and a target positive control were extracted in the same way as the clinical samples to evaluate the whole process. Levey-Jennings graphs demonstrated that 10/13 (76.9%) concentration values (in copies/μL) of the target positive control of extraction were within ±1SD and 3/13 (23.1%) were within ±2SD (Figure 2a). When normalization ratio was used as control value of the extraction, 11/13 (84.6%) values were within ±1SD as it was shown in Levey-Jennings graphs (Figure 2b).

### 3.2. cfDNA Quantification

Before mutation analysis, total cfDNA quantification was performed for all samples with IDQUANTd kit (ID-Solutions) to evaluate their quality and their concentration levels. Based on these values, every sample was subjected to appropriate dilution, if needed, to a final DNA input of 400 to 5000 copies per PCR to achieve levels of sensitivity ranging from 1% to 0.1% for the mutation analysis with crystal dPCR.

**Figure 3:**
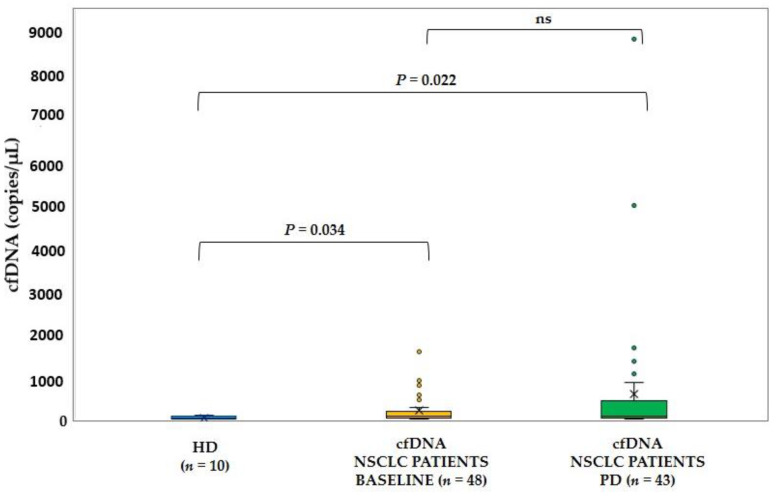
Boxplots demonstrating the differences in total cfDNA concentration (copies/μL) between healthy donors’ samples (*n* = 10, NSCLC patient samples at baseline (*n* = 48) and at PD (*n* = 43).

The mean value of cfDNA concentrations for HDs samples was 52.8 copies/μL (median: 41.8, range: 27.2–101 copies/μL) whereas the mean value of cfDNA concentration for patient samples at baseline was 197 copies/μL (median: 82.8, range: 13.6–1579 copies/μL) and at PD was 589 copies/μL (median: 84.8, range: 19.5–8822 copies/μL). There was a statistically significant difference between HD group and NSCLC patients’ group at baseline (Mann-Whitney test, *p* = 0.034) or at PD (Mann-Whitney test, *p* = 0.022), respectively. (Figure 3). No statistically significant difference was observed between the two time points (Mann-Whitney test, *p* = 0.592).

**Figure 4:**
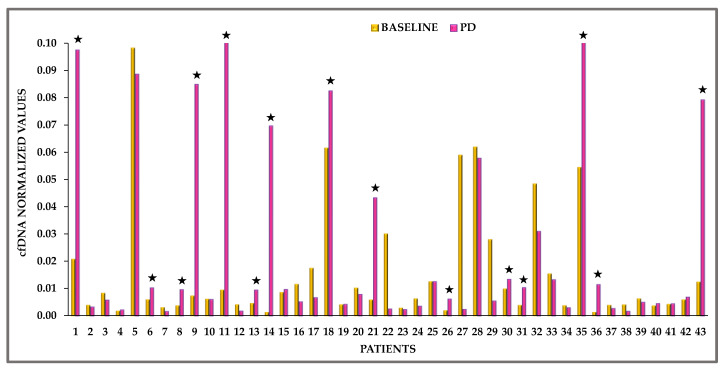
cfDNA fluctuations in plasma samples between baseline and PD for 43 patients based on normalized cfDNA values, ★: cases where normalized cfDNA values are higher in PD than at the baseline.

Differences in normalized cfDNA values between time points for the same patient are indicative for the evolution of its pathological state regarding treatment efficacy. Such differences between baseline (*n* = 43) and PD samples (*n* = 43) were evaluated for the studied cohort of patients. As can be seen in Figure 4, 15/43 (34.9%) patients had higher levels of normalized cfDNA values at PD compared to matched baseline samples.

### 3.3. EGFR Mutation Analysis in cfDNA with Crystal Digital PCR

91 plasma samples of 48 patients were analyzed for the detection of EGFR mutations in plasma cfDNA with crystal dPCR at two different time points: (a) baseline (*n* = 48) and (b) PD (*n* = 43).

Exon 19 and exon 21 sensitizing mutations: 26/91 (28.6%) samples were found positive for exon19 deletions. 14/48 (29.2%) were found positive in baseline samples and 12/44 (27.3%) at PD. For exon 21, 15/91 (16.5%) were positive for L858R mutation and 3/91 (3.3%) for the L861Q mutation. More precisely, at baseline samples 7/48 (14.6%) were positive for L858R and 2/48 (4.2%) for L861Q whereas at PD samples 8/44 (18.2) were positive for L858R only one for L861Q.

Exon 20 resistance mutations: 14 out of 91 (15.4%) samples were positive for the resistance mutation T790M. At baseline samples, T790M was found positive for 13 out of 48 (27.1%) samples and at PD only in one sample that was also positive for the presence of C797S in cis with T790M.

Exon 18 and exon 20 rare mutations: Exon 18 G719X mutation was detected in parallel with Exon 20 S768I in five out of 91 (5.5%) samples. Three out of 48 (6.2%) samples and two out of 43 (4.6%) samples were positive for both mutations at baseline and PD, respectively.

### 3.4. Evaluation of MAFs in EGFR Mutations between Baseline and PD

**Figure 5:**
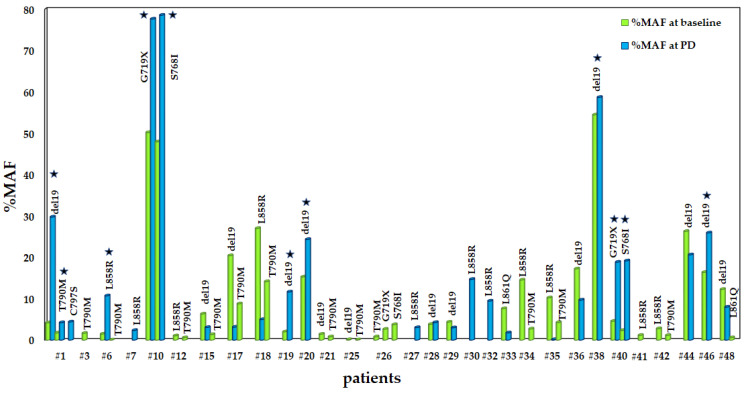
Evaluation of %MAF of EGFR mutations at baseline and at PD, ★: cases where %MAF are higher in PD than at the baseline.

We compared the % MAFs of each type of EGFR mutation in plasma between baseline and PD. In some patients, mutations disappear at PD and in some patients are maintained or reemerge. For the patients harboring exon 19 deletions, 12/14 (85.7%) maintained the mutation whereas two of them lost it. Regarding the exon 21 L858R mutation, 3/12 maintained the mutation, in 4/12 it was present only at baseline and in 5/12 it was detected only at PD. The resistance mutation T790M was lost at PD in all cases except for one that was found concomitantly with C797S. One patient lost exon 21 L861Q whereas another one maintained the mutation at PD. As for the compound mutations G719X and S768I, two patients maintained both mutations at PD and one was found negative. The differences in %MAF between these two time points for every type of mutation are depicted in Figure 5. As can be seen in Figure 5, in eight patients (#1, #6, #10, #19, #20, #38, #40, #46) %MAFs for various EGFR mutations were significantly increased at PD.

**Figure 6:**
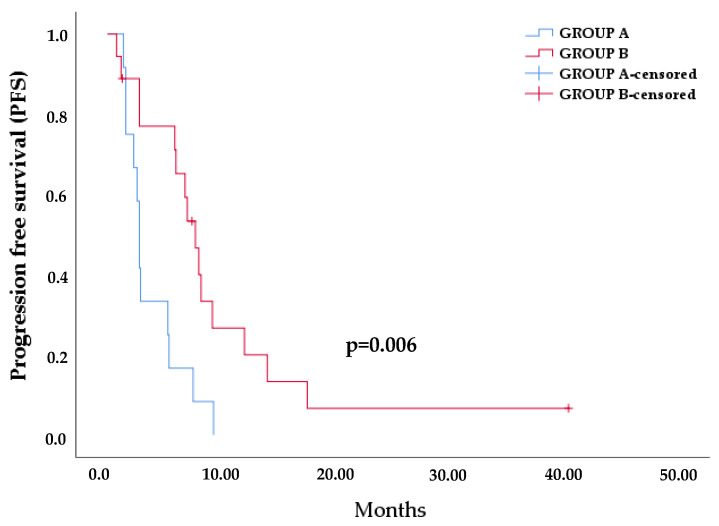
Difference in PFS depending on %MAFs before treatment with osimertinib and at PD between group A and group B.

Furthermore, we divided the patients into two groups: (A) those that presented an increase of %MAFs of different EGFR mutations at PD or presented EGFR mutations only at PD and (B) those who either lost EGFR mutations at PD or presented lower %MAFs. We estimated the differences between the two groups in terms of PFS and observed that the first group progressed earlier (mean PFS: 3.8 months) than the second one (mean PFS: 9.8 months) with a statistically significant difference (log rank: *p* = 0.006) (Figure 6). 

### 3.5. Direct Comparison of Crystal Digital PCR and the FDA-Approved Cobas^®^ EGFR Mutation Test V2

**Figure 7:**
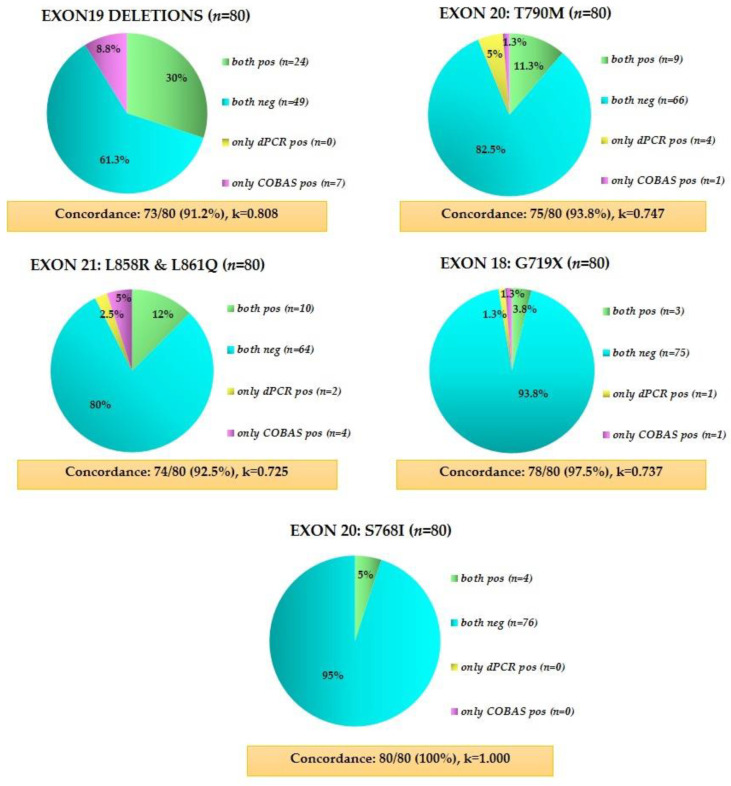
Direct comparison of EGFR mutations detected with crystal dPCR and FDA-approved cobas^®^ EGFR mutation test v2.

In parallel with crystal dPCR analysis, we performed EGFR mutation analysis in 80 identical matched plasma samples using the FDA-approved cobas^®^ EGFR mutation test v2. The concordance rates between the two EGFR mutation detection methods were high for all types of mutations as described in detail in Figure 7.

### 3.6. Comparison between Primary Tissue and Plasma cfDNA for EGFR Mutation Genotyping at Baseline

All patients were subjected to tissue biopsy prior to treatment with osimertinib. A direct comparison of EGFR genotyping between the primary tissue and baseline plasma cfDNA samples revealed high rates of concordance for the mutations in the different exons of the EGFR gene. For the exon 19 deletions and exon 20 T790M mutation the agreement is defined as moderate (Cohen’s kappa index: 0.455 and 0.395, respectively). All results are summarized in Table 1. As can be seen in Table 1, in total, for all EGFR mutations, 29 samples were positive in the primary tissue but negative in cfDNA.

### 3.7. EGFR Mutation Detection in CTC-Derived gDNA Using Crystal dPCR

**Figure 8:**
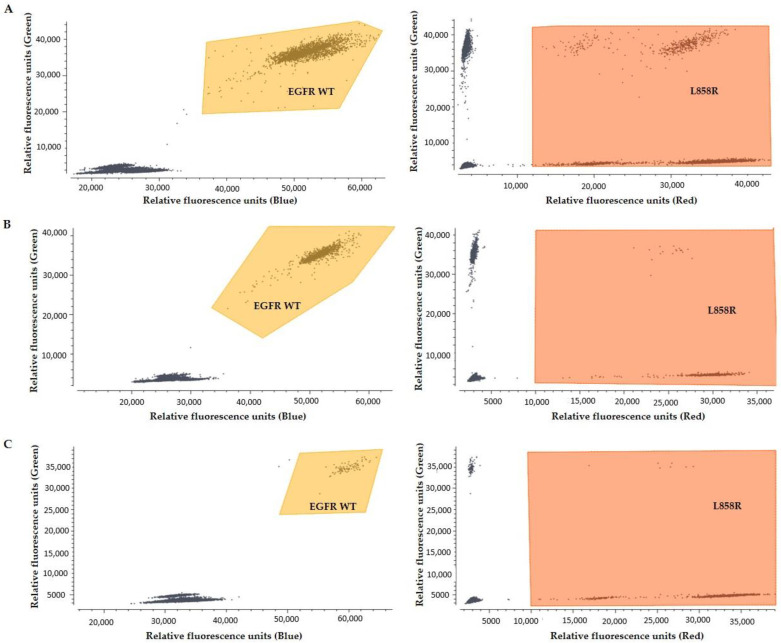

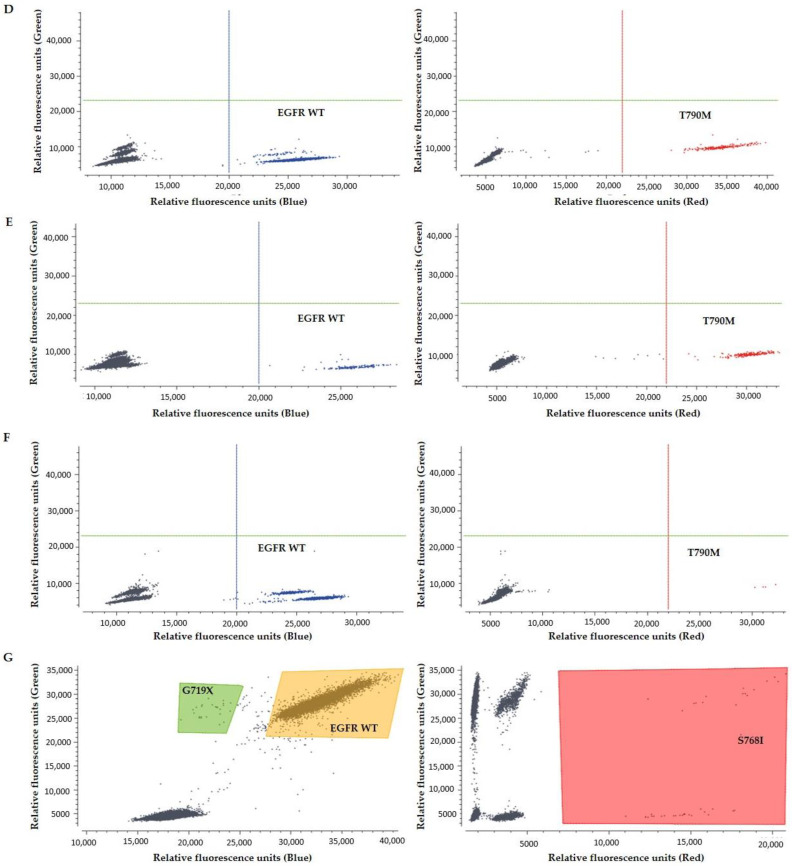
Characteristic crystal dPCR plots for EGFR mutations: (**A**) L858R in NCI-H1975 cell line derived gDNA, (**B**) L858R in cfDNA sample (Pt #18) before and (**C**) after (**C**) WGA, (**D**) T790M in cfDNA sample (Pt #18) before and (**E**) after WGA, (**F**) T790M in CTC- derived gDNA (Pt #12) after WGA and (**G**) G719X and S768I in CTC- derived gDNA (Pt #10) after WGA.

The human cancer cell line NCI-H1975 was used to validate the protocol of EGFR mutation detection in gDNA derived from CTC fractions. For the establishment and evaluation of the protocol 100 NCI-H1975 cells were prepared and spiked into 10 mL PB of HD, enriched by Parsortix™, subjected to WGA and then were further analyzed for the detection of exon 21 L858R mutation by crystal dPCR. As can be seen in Figure 8A L858R could be clearly detected in this cell line derived DNA. Moreover, in the same cell free DNA samples EGFR mutations were detected before and after WGA: L858R (Figure 8B,C), T790M (Figure 8D,E). In CTCs EGFR mutations were detected after WGA (Figure 8F,G).

**Figure 9:**
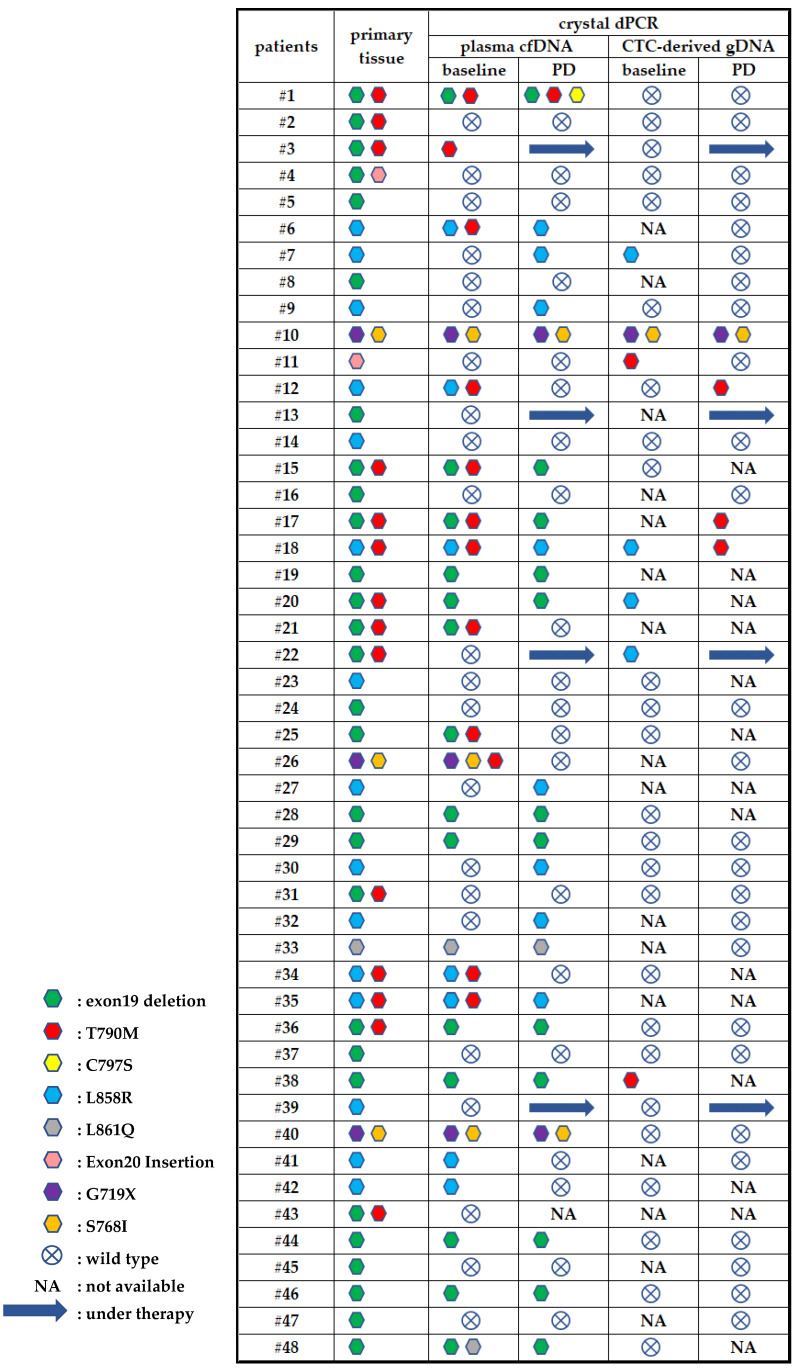
Direct comparison of EGFR mutations detected in primary tissue, plasma cfDNA and CTC-derived gDNA.

A total number of 64 matched CTC-derived gDNA samples were analyzed for the detection of EGFR mutations using crystal dPCR; 11 samples were found positive for EGFR mutations (Figure 9) with %MAF ranging from 0.2 to 2.25%. At baseline four samples were found positive for L858R, two were positive for T790M and one positive for the compound mutations G719X and S768I. At PD, T790M was detected in three samples and G719X and S768I in one sample. In Pt #10 and Pt #18 EGFR mutations detected in CTC baseline samples were in accordance with the mutations found in the corresponding plasma cfDNA and primary tissue. In Pt #7, L858R was detected in the CTC-derived gDNA at the baseline sample whereas in the corresponding plasma cfDNA was absent. Pt #20 and Pt #22 were positive for L858R, and Pt #11 and Pt #38 were positive for T790M at baseline CTC-derived gDNA samples but negative in the primary tissue and cfDNA plasma samples. Regarding CTC samples at PD, Pt #10 matches with cfDNA result whereas in three patients (#12, #17, #18) T790M was only detected in CTC samples. Among 48 patients tested, only four are still under osimertinib therapy without progression of disease. Three out of these four patients were negative for EGFR mutations in plasma cfDNA and corresponding CTCs at baseline.

## 4. Discussion

The management of EGFR mutant NSCLC has successfully switched from chemotherapy to targeted treatment improving substantially the survival outcomes of NSCLC patients [39]. Osimertinib is widely approved as one of the most effective EGFR TKIs when administered either as 1st or 2nd line treatment [39]. ctDNA analysis for longitudinal monitoring of NSCLC patients has already entered the clinical setting [14] but technical challenges of analysis are still remaining [40]. Emerging technologies such as dPCR have changed the molecular biomarker analysis setting by providing high sensitivity, accuracy, and precision. In NSCLC, dPCR assays have demonstrated clinical utility in detecting EGFR mutations [41] with comparable results to next generation sequencing (NGS) [42]. cfDNA analysis requires highly sensitive detection methods to track rare genetic aberrations of disease that may guide treatment as it was already shown in EGFR TKIs [43,44,45,46].

The scope of the present study was to evaluate the presence and estimate mutation allele frequencies of EGFR mutations in plasma cfDNA and corresponding CTC-derived gDNA with crystal dPCR in a group of EGFR mutant NSCLC patients under osimertinib therapy before treatment initiation and at progression of disease. In this study, we followed in detail all the proposed guidelines both at the preanalytical and analytical level and implemented all required quality control steps before EGFR mutation analysis.

Before cfDNA extraction, a specified amount of an exogenous internal control was added in every single plasma sample including negative, positive control and clinical samples. Following the cfDNA isolation from plasma, we simultaneously quantified the concentrations of total cfDNA of each sample and of the exogenous internal control. Levey-Jennings graphs clearly showed that most of our positive control values either presented as concentrations or as normalized DNA values were within ±1SD indicating a very good performance of the whole analytical procedure. Absolute DNA quantification allowed us to calculate the proper number of cfDNA copies for EGFR mutation analysis and thus we could achieve the maximum sensitivity of the crystal dPCR assays, ranging from 1% to 0.1%. 

Several factors affect cfDNA levels in healthy population and cancer patients. During proliferation and metastasis, levels of cfDNA in the bloodstream of cancer patients reflect the increased activity of apoptosis, necrosis and secretion and might be indicative of disease progression [47,48]. In this study, we compared the cfDNA levels of HDs and NSCLC patients at baseline and at PD and report a statistically significant difference between the three groups as expected. Furthermore, we compared the plasma cfDNA normalized values during osimertinib therapy between baseline and PD. The normalization ratio was used to standardize inter-assay fluctuations for the same patient during longitudinal monitoring. In 15/43 (34.9%) PD samples, the total cfDNA levels were significantly higher than matched baseline samples demonstrating an indicative correlation with disease progression.

When we compared EGFR mutation rates detected by crystal dPCR in 80 identical plasma samples from both time points with the FDA approved cobas^®^ EGFR Mutation test v2 as a reference method, the overall concordance between the two assays was strong for all EGFR mutations tested and the agreement evaluated by Cohen’s kappa ranged from substantial to almost perfect, in accordance with previous studies [49]. The discrepancies that were observed between the two assays for exon 19 deletions and exon 20 resistance mutation C797S might be explained by the detection capacity of the two kits. In case of T790M, crystal dPCR detected more positive events than the cobas^®^ assay, indicating the necessity of using highly sensitive methodologies to detect rare but significant events that are crucial for treatment monitoring and consulting [50].

Differences in plasma EGFR genotyping between baseline and PD revealed the reemergence or maintenance of EGFR mutations at PD. In some cases, a distinctively higher %MAF of various EGFR mutations were detected in plasma cfDNA of patients at PD compared to those at baseline. Survival analysis between those patients that presented higher %MAFs at PD and those that lost EGFR mutations after osimertinib therapy revealed a statistically significant difference between the two groups of patients indicating the aggressiveness of disease that led earlier to PD. However, another important observation is that in almost all cases T790M detected before initiation of treatment was lost during PD, clearly indicating that this clone has disappeared after osimertinib therapy. Previous evidence has demonstrated that patients under osimertinib who lost T790M presented shorter time to treatment discontinuation (TTD) in comparison with those that maintained it [51]. Only in one patient crystal dPCR had successfully identified the presence of C797S mutation in *cis* configuration with T790M during PD. The acquired C797S mutation is one of the most prevalent resistance mechanisms during disease progression with osimertinib [51,52]. The identification of its *cis* or *trans* configuration in relation to T790M mutation is an important information that could define the proper subsequent treatment options [53]. 

By comparing the presence of EGFR mutations detected in plasma cfDNA with those already detected in primary tissue we found a concordance ranging from 70.8 to 100% for all EGFR exons. There were some discrepancies that affect the agreement of these two types of EGFR genotyping; exon 19 deletions in the primary tissue were detected at a higher percentage than in plasma cfDNA whereas exon 20 T790M was solely detected either in plasma cfDNA by crystal dPCR or in primary tissue for some patient samples. Discordances between tissue and plasma genotyping are frequently observed and one possible reason could be the sensitivity of different methodologies used [50]. However, NSCLC tumors represent high spatial and temporal diversity regarding their mutational profiles [54,55]. Hence, a single tumor biopsy may not be a dynamic representative of the current mutation burden. 

To the best of our knowledge, this the first study conducted in a group of NSCLC patients under osimertinib therapy that combines the detection of EGFR mutations in plasma cfDNA and CTCs with the technology of crystal dPCR. Despite the small number of samples found positive for EGFR mutations in CTC fractions, direct comparison with plasma cfDNA and primary tissue revealed interesting results. In three cases (Pt #7, Pt #10, Pt #18), EGFR mutations in CTC fractions at baseline matched with those in primary tumor, an observation that is in accordance with previous studies [21,22,23,24,56]. In two patients (Pt #11, Pt #38), T790M was detectable in CTC-derived gDNA at baseline but not in the corresponding plasma cfDNA or in the primary tissue sample. Interestingly, both patients had a significantly low PFS (2.5 and 1.6 months, respectively); we hypothesize that this may be a case of subclonal T790M, potent enough to lead to rapid progression of disease as it was clearly demonstrated very recently in a study including patients from AURA3 phase III trial [57]. Three other patients (Pt #12, Pt #17, Pt #18) were found positive for T790M in CTC-derived gDNA samples at PD. However, according to cfDNA plasma genotyping, the current mutation was lost at this time. Discordances between cfDNA in plasma and CTCs may be indicative of tumor heterogeneity that characterizes NSCLC [58] and also predictive for the resistance mechanisms that occur under selective therapy pressure due to the dominance of T790M wild type clones as it was previously described [59]. Comprehensive analysis of EGFR mutations in cfDNA and CTCs could be more informative regarding the treatment monitoring of NSCLC patients as it was recently demonstrated in studies that included both liquid biopsy biomarkers [60,61].

## 5. Conclusions

Many preanalytical and technological issues need to be considered and overcome to move faster towards the clinical utility of liquid biopsy. In this sense, extremely sensitive methodologies such as crystal dPCR allowed us to track tumor evolution through the detection of low abundance mutations in cfDNA and CTC fractions predictive for the treatment outcomes of NSCLC patients under osimertinib. In our study, crystal dPCR exhibited high concordance rates in correlation with the FDA-cleared cobas technology; however, in some cases crystal dPCR was more sensitive in detecting the T790M mutation which is the key resistance mutation found during treatment with 1st and 2nd EGFR TKIs. The presence of EGFR mutations in paired CTC-derived gDNA analyzed for the first time in a group of NSCLC patients under osimertinib and the discrepancies found between CTC fractions and tumor or cfDNA genotyping, confirmed previous evidence about spatial and temporal tumor heterogeneity and clonal evolution. Crystal dPCR combines the unique benefits of sensitivity and accuracy with the multiplexing capacity of the three detection channels for the detection of multiple EGFR mutations in plasma cfDNA samples and corresponding CTCs of NSCLC patients.

## Figures and Tables

**Table 1:** Direct comparison between EGFR mutations in plasma cfDNA at baseline using crystal dPCR and corresponding primary tissue.

**EXON 19 del dPCR** **(ctDNA)**				
EXON19del tissue		**-**	**+**	Total
	-	20	0	20
	+	14	14	28
	total	34	14	48
	Concordance:	34/48 (70.8%)	k = 0.455	
**EXON 20 T790M dPCR** **(ctDNA)**				
EXON20 T790M tissue		-	+	total
	-	28	6	34
	+	6	8	14
	total	34	14	48
	Concordance:	36/48 (75%)	k = 0.395	
**EXON21 dPCR** **(ctDNA)**				
EXON21 tissue		-	+	total
	-	32	0	32
	+	7	9	16
	total	39	9	48
	Concordance:	41/48 (85.4%)	k = 0.632	
**EXON18 G719X dPCR** **(ctDNA)**				
EXON18 G719X tissue		-	+	total
	-	45	0	45
	+	0	3	3
	total	45	3	48
	Concordance:	48/48 (100%)	k = 1.000	
**EXON20 RARE dPCR** **(ctDNA)**				
EXON20 RARE tissue		-	+	total
	-	43	0	43
	+	2	3	5
	total	45	3	48
	Concordance:	46/48 (95.8%)	k = 0.729	

## Data Availability

The data presented in this study are available on request from the corresponding author. The data are not publicly available due to ethical restrictions.

## Supplementary Materials

The following is available online at https://www.mdpi.com/article/10.3390/cancers13112736/s1, Table S1: Differences between cobas^®^ EGFR mutation test and ID-solutions kits.
