# Supplementary material for: Detection of EGFR Mutations in Plasma cfDNA and Paired CTCs of NSCLC Patients before and after Osimertinib Therapy Using Crystal Digital PCR
Source: Cancers (Basel). 2021 May 31;13(11):2736. doi: 10.3390/cancers13112736 (PMC8197887; doi:10.3390/cancers13112736)
Supplement: Supplementary file 1 [file cancers-13-02736-s001.zip › cancers-1192044-supplementary.pdf]

Supplementary Table S1: Differences between cobas® EGFR mutation test and ID-solutions kits

EGFR Mutation group	EGFR nucleic acid sequence	COSMIC ID	cobas EGFR test	ID-solutions tests
EXON 18 G719X	c.2156 G>C	6239	×	×
	c.2155 G>A	6252	×	×
	c.2155 G>T	6253	×	×
	c.2154-2155 GG>TT	18441		×
	c.2156 G>A	18425		×
EXON19 DELETIONS	c.2240_2251del12	6210	×	×
	c.2239_2247del9TTAAGAGAA	6218	×	×
	c.2238_2255del18	6220	×	×
	c.2235_2249del15	6223	×	×
	c.2236_2250del15	6225	×	×
	c.2239_2253del15	6254	×	
	c.2239_2256del18	6255	×	×
	c.2237_2254del18	12367	×	×
	c.2240_2254del15	12369	×	×
	c.2240_2257del18	12370	×	×
	c.2239_2248TTAAGAGAAG>C (complex)	12382	×	×
	c.2239_2251>C(complex)	12383	×	×
	c.2237_2255>T(complex)	12384	×	×
	c.2235_2255>AAT	12385	×	
	c.2237_2252>T	12386	×	×
	c.2239_2258>CA(complex)	12387	×	×
	c.2239_2256>CAA	12403	×	×

	c.2237_2253>TTGCT	12416	×	
	c.2238_2252>GCA(complex)	12419	×	×
	c.2238_2248>GC(complex)	12422	×	×
	c.2237_2251del15	12678	×	×
	c.2236_2253del18	12728	×	×
	c.2235_2248>AATTC	13550	×	
	c.2235_2252>AAT(complex)	13551	×	×
	c.2235_2251>AATTC	13552	×	
	c.2253_2276del24	13556	×	
	c.2237_2257>TCT	18427	×	
	c.2238_2252del15	23571	×	×
	c.2233_2247del15	26038	×	
	c.2236_2253> ACG	/		×
	c.2239_2250>CCC	/		×
	c.2239_2261>CAATT	/		×
	c.2238_2261>GCAACATCT	/		×
	c.2238_2256>GCAA	/		×
	c.2236_2248>CAAC	13557		×
	c.2236_2253>CAA	22999		×
	c.2239_2256del18	6255		×
EXON 20 S768I	c.2303G>T	6241	×	×
EXON20 INSERTIONS	c.2307_2308ins9GCCAGCGTG	12376	×	×
	c.2319_2320insCAC	12377	×	×
	c.2310_2311insGGT	12378	×	×
	c.2311_2312ins9GCGTGGACA	13428	×	×

	c.2309_2310AC>CCAGCGTGGAT	13558	×	×
EXON 20 T790M	c.2369C>T	6240	×	×
EXON 21 L858R	c.2573T>G	6224	×	×
	c.2573_2574TG>GT	12429	×	×
EXON 21 L861Q	c.2582T>A	6213	×	×
EXON 21 C797S	c.2389T>A	6493937		×
	c.2390G>C	5945664		×
